# Integrative Analysis of Volatile Flavor Compounds and Transcriptome Reveals Underlying Mechanisms Linked to Fatty Acid Content in Dabieshan Cattle

**DOI:** 10.3390/foods15081423

**Published:** 2026-04-19

**Authors:** Liu Zhang, Qian Li, Hai Jin, Shuanping Zhao, Huibin Zhang, Xinyi Du, Qinggang Li, Lei Xu

**Affiliations:** 1 Anhui Provincial Key Laboratory of Livestock and Poultry Product Safety, Institute of Animal Husbandry and Veterinary Medicine, Anhui Academy of Agricultural Sciences, Hefei 230031, China; 2 College of Animal Science, Anhui Science and Technology University, Chuzhou 233100, China

**Keywords:** transcriptomics, Dabieshan cattle, fatty acid, volatile flavor compounds, longissimus dorsi

## Abstract

To investigate the associations between genes involved in fatty acid composition and volatile flavor compounds (VOCs), Dabieshan (DBS) cattle were selected and stratified into high (H: 0.018–0.024 g) and low (L: 0.007–0.012 g) groups according to the fatty acid content in the longissimus dorsi (LD). Integrated analysis using two-dimensional gas chromatography–time-of-flight mass spectrometry (GC×GC-TOF-MS) and transcriptomics systematically revealed differences in VOCs and gene expression profiles, along with their associations with fatty acid composition. The relative contents of aldehydes, esters, and hydrocarbons were significantly higher in the group H, whereas the group L exhibited elevated levels of alcohols, acids, and heterocyclic compounds. Among 54 differentially abundant VOCs identified, (E)-2-Nonenal (ROAV = 100) was established as the key flavor contributor. Transcriptomic analysis identified 678 differentially expressed genes (DEGs), with eight candidate genes implicated in fatty acid composition pinpointed through GO and KEGG enrichment analyses. Further correlation analysis showed that the expression levels of SGPL1, KLF15 and SLC27A6 were significantly correlated with the contents of polyunsaturated fatty acids (C22:5n-3, C18:3n-3, C18:2n-6, C18:1n-9c). There was also a significant correlation between the above fatty acids and characteristic flavor compounds including 3-Hexanone, (E)-2-Nonenal, (E,E)-2,4-Octadienal and Butanal. This study suggested potential links among fatty acid composition, key genes and characteristic flavor compounds in Dabieshan cattle, providing new insights into the genetic improvement of flavor quality of local cattle breeds.

## 1. Introduction

The flavor characteristics, quality, and nutritional value of meat are significantly influenced by its fatty acid composition [1,2]. According to the saturation level of their hydrocarbon chains, fatty acids can be classified into three types: saturated fatty acids (SFAs), monounsaturated fatty acids (MUFAs), and polyunsaturated fatty acids (PUFAs). Excessive intake of foods containing SFAs may increase cholesterol levels in human blood lipoproteins, thereby increasing the risk of various cardiovascular diseases, whereas unsaturated fatty acids (UFAs) can mitigate this risk [3].

The characteristic flavor of beef during thermal processing originates from free radical chain reactions triggered by fatty acid oxidation, with the resulting volatile compounds forming the basis of the flavor matrix [4,5]. The specific fatty acid precursors largely determine the flavor quality, as both saturated and UFAs undergo cleavage during thermal oxidation to generate volatile substances including aldehydes, alcohols, ketones, hydrocarbons, esters, ethers, and furans [6,7,8]. Yu et al. demonstrated that C18:2n-6, C20:4n-6, and DHA-phosphatidylcholine generated active flavor compounds with fatty and roasted aromas through oxidation [9]. Song et al. identified (E,E)-2,4-Decadienal and (E)-2-Nonenal as key aroma compounds in oxidized beef, owing to their low odor thresholds and high aromatic activity [10]. The formation of flavor in yak meat was shown to be closely associated with the dynamic equilibrium of metabolites, including stearic acid and glucose-6-phosphate [11]. The metabolic and genetic mechanisms underlying fatty acid composition are suggested to be critical for improving meat quality; however, elucidating these mechanisms poses significant challenges and incurs high costs. Transcriptome technology enables systematic dissection of this regulatory network, offering considerable potential for enhancing beef flavor and overall quality [12]. Schettini et al. discovered that differentially expressed genes (*ECHS1*, *IVD*) and hub genes (*ASB5*, *ERLIN1*) in Nellore cattle significantly affect the deposition of essential fatty acids by regulating the metabolism of linoleic acid and alpha-linolenic acid [13]. Gai et al. employed multi-omics techniques to identify four key metabolic pathways influencing the flavor formation of Beijing-You chicken breast muscle. They also screened important metabolites and regulatory genes affecting meat quality and flavor, including glycine, serine, glutamate, threonine, *LPC*, *IMP*, *CBS*, *GATM*, *GAD2*, *PNPLA6*, *ITAE*, and *AMPD1* [14]. In a study on Guangdong small-ear spotted pigs, Wang et al. discovered that the *AMD1* gene regulates the polyamine metabolic pathway, thereby altering p-cresol levels. This process is considered a critical molecular mechanism contributing to the unique flavor characteristics of this pig breed [15].

DBS cattle, an outstanding indigenous breed in China, demonstrate exceptional disease resistance, remarkable tolerance to rough feeding, a rich flavor, and superior meat quality [16]. They are mainly found in the Dabie Mountains and the middle and lower reaches of the Yangtze River basin [17,18]. Owing to prolonged genetic isolation from other breeds, they have developed a unique genetic background, which offers significant potential for the targeted improvement of meat quality traits. However, there remains a lack of systematic and in-depth research on the molecular mechanisms related to fatty acid composition characteristics of DBS cattle muscle and their association with flavor formation. Multi-omics technologies, with their capability to systematically analyze genetic regulatory networks and metabolic pathways, have emerged as powerful tools for elucidating the complex regulatory mechanisms of meat quality [19,20]. Therefore, this study focused on DBS cattle. We collected longissimus dorsi (LD) muscle samples to analyze the composition and content of fatty acids using quantitative detection technology. Based on the extreme differences in fatty acid content, the samples were categorized into high- and low-content groups. The primary objectives of this study were to elucidate the genetic basis of meat quality traits in DBS cattle, to identify candidate genes associated with fatty acid composition, and to explore the relationship between fatty acid levels and meat quality.

## 2. Materials and Methods

### 2.1. Animal Sample Collection

The experimental samples in this study were obtained from 20 DBS cattle, each with a body weight of approximately 350 kg, all of which were raised at Taihu County Jiuhong Agricultural Comprehensive Development Co., Ltd. (Anqing, China) These cattle were weaned at six months of age and fattened under identical feeding and management conditions until 24 months of age. All individuals were healthy, well-developed, and subjected to 24 h fasting and 8 h water deprivation prior to slaughter. Following industry slaughter standards, the cattle were weighed and stunned by electrical shock, followed by exsanguination and removal of the internal organs, head, hooves, skin, and tail. After slaughter, the LD between the 12^th^ and 13^th^ ribs of each animal was collected. LD samples for RNA extraction were placed in RNAlater® TissueProtect Tubes (Qiagen, Hilden, Germany), flash-frozen in liquid nitrogen, and stored at −80 °C. Meanwhile, LD samples for fatty acid composition analysis were vacuum-packed and stored at −80 °C.

Finally, among the 20 individuals with consistent genetic backgrounds, the four with the highest and four with the lowest fatty acid content were selected to form the H group (*n* = 4) and L group (*n* = 4), respectively, corresponding to an extreme sampling approach of the upper and lower 25%.

### 2.2. Fatty Acid Determination

Following a modified protocol based on Hoving et al. [21], the fatty acid composition was analyzed. The procedure commenced with the homogenization of a 0.5 g sample together with 1 mL of chloroform–methanol (2:1). This was done using a high-throughput tissue grinder set to 60 Hz for 1 min, and the step was repeated twice. A 30 min ultrasonication at room temperature was then applied to the mixture. Centrifugation at 12,000× *g* for 5 min at 4 °C yielded a homogenate supernatant, which was transferred. For methylation, 2 mL of 1% sulfuric acid–methanol solution was introduced, and the mixture was vortexed for 1 min. It was then incubated at 80 °C for 30 min in a water bath. Once cooled, extraction of fatty acid methyl esters was initiated by adding 1 mL of n-hexane. The tube was vortexed for 30 s and left to stand for 5 min. To wash the organic phase, 5 mL of ice-cold ultrapure water was added, and the mixture was centrifuged at 3500× *g* for 10 min at 4 °C. The subsequent step involved transferring 700 μL of the upper n-hexane layer to a fresh 2 mL tube containing 100 mg of anhydrous sodium sulfate for dehydration. After vortexing for 30 s, the tube was centrifuged at 12,000× *g* for 5 min. A 20-fold dilution with n-hexane was performed on the sample, from which a 300 μL aliquot was taken into another 2 mL tube. Here, 15 μL of 500 mg/L methyl salicylate (internal standard) was added, and the mixture was vortexed briefly for 10 s. The final preparation step involved transferring 200 μL of the resulting supernatant to a GC-MS vial for subsequent analysis.

A Thermo Trace 1300 gas chromatography system (Thermo Fisher Scientific, Waltham, MA, USA) coupled to a Thermo ISQ 7000 mass spectrometer (Thermo Fisher Scientific, Waltham, MA, USA) was employed for the quantitative analysis of fatty acid methyl esters. Operating in split mode, the analysis used a 1 μL injection volume, and the injector was set to 250 °C. The column temperature was programmed with an initial hold at 80 °C for 1 min. It was then raised at 20 °C/min to 160 °C and held for 1.5 min, followed by an increase to 196 °C at 3 °C/min with an 8.5 min hold. The final stage involved ramping the temperature to 250 °C at 20 °C/min and maintaining it for 3 min. Identification of individual fatty acids was achieved qualitatively by comparing sample fatty acid methyl ester retention times against those of a commercially available 46-component mixed standard (NU-CHEK-PREP, Shanghai, China).

### 2.3. Determination of Volatile Flavor Compounds

A GC×GC-TOF MS system (Agilent Technologies, Palo Alto, CA, USA) was utilized for the determination of volatile compounds in beef. The analytical protocol, adapted from the method described by Monica and Pavlidis [22,23], is detailed below. Preceding SPME extraction, the fiber underwent a 10 min conditioning step at 270 °C. For sample preparation, 1.0 g of beef was weighed into a 20 mL headspace vial. After adding 10 μL of deuterated n-hexanol-d13 internal standard solution (C/D/N Isotopes) and sealing the vial, volatile release was facilitated by incubating the sample at 100 °C for 30 min. Subsequently, the conditioned SPME fiber was exposed to the vial headspace at 100 °C for 40 min to adsorb analytes. Thermal desorption of the adsorbed compounds was achieved by inserting the fiber into the GC inlet, set at 250 °C for 5 min. Post-injection, the fiber was re-conditioned under the same initial parameters. A separate 10 μL aliquot of n-alkane standard was processed similarly in a 20 mL headspace vial for calibration. The injection was performed in splitless mode with the injector temperature at 250 °C and a 5 min desorption time. A constant helium flow of 1.0 mL/min was used as the carrier gas. The GC oven program for the primary DB-Heavy Wax column commenced at 40 °C (hold for 3 min), increased to 50 °C at 3 °C/min, then to 120 °C at 6 °C/min, followed by a ramp to 220 °C at 5 °C/min, and a final hold for 15 min. The secondary Rxi-5Sil MS column temperature was programmed to be 5 °C higher throughout. The modulator operated at a temperature 15 °C above the secondary column, with a period of 6.0 s. The inlet was maintained at 250 °C. MS data were collected at an acquisition rate of 200 spectra/s under 70 eV electron ionization, scanning from *m*/*z* 35 to 550.

Flavor compounds were annotated by matching the mass spectral data against the NIST2020 database using Chroma TOF software Version 4.22. Quantification was performed using the peak area normalization method, and the relative content of each compound was expressed as the percentage of its peak area relative to the total peak area. Chemical classification and characterization of flavor compounds were carried out using the PubChem database (https://pubchem.ncbi.nlm.nih.gov) and Classyfire software Version 4.1 [24]. The relative odor activity value (ROAV) method was employed to evaluate the flavor profile of the samples, and the calculation of ROAV was based on the method described by Zhu et al. [25]. Multivariate statistical analysis was performed using the SIMCA-P Version 13.0 software package and the ropls package in R Version 4.4.0. [26]. Differential VOCs were screened based on a *p*-value < 0.05 and a VIP > 1. Additionally, a network tree diagram of flavor compounds was constructed using the igraph Version 1.3.1 package based on the Favordb database to elucidate their unique sensory flavor characteristics [27].

### 2.4. RNA-Seq Library Preparation

The extraction of total RNA from LD tissue was performed utilizing TRIzol reagent (Invitrogen, Carlsbad, CA, USA). To assess the obtained RNA, its concentration, purity, and integrity (RIN) were determined using the Qubit RNA Assay Kit (Thermo Fisher Scientific, Waltham, MA, USA), NanoPhotometer nucleic acid quantifier (Implen GmbH, Munich, Germany), and Agilent Bioanalyzer 2100 RNA 6000 Nano chip (Agilent Technologies, Santa Clara, CA, USA), respectively. Additionally, denaturing agarose gel electrophoresis served as a further verification step for RNA quality. The construction of eight cDNA libraries was achieved by reverse transcribing the qualified samples with the mRNA-Seq Sample Preparation Kit (Illumina, San Diego, CA, USA). Subsequent paired-end sequencing (2 × 150 bp) was conducted utilizing the Illumina Novaseq 6000 platform (LC-Bio Technology Co., Hangzhou, China). For initial processing, quality control filtering of the raw sequencing data was performed with fastp software Version 0.23.4 [28]. Adapter trimming, removal of low-quality reads, and sequences with N base proportions exceeding 10% were performed using Trimmomatic Version 0.39 to obtain high-quality clean reads. Alignment of the clean reads against the bovine reference genome (ARS-UCD1.2) was performed employing HISAT2 Version 2.2.1 software [29]. Based on the alignment results, transcript assembly was performed using StringTie Version 2.2.1 [30], and gene expression levels were quantified using RSEM Version 1.3.3 [31]. Gene expression levels were expressed as Transcripts Per Million. Differentially expressed genes (DEGs) between groups H and L were identified using the edgeR R package [32], with selection criteria set at |log_2_FC| > 1 and a *p*-value < 0.05. For functional annotation, these DEGs were analyzed for GO and KEGG pathways using the DAVID database (https://davidbioinformatics.nih.gov). The OmicShare platform (https://www.omicshare.com) was then utilized to generate related visualizations.

### 2.5. Integrated Multi-Omics Analysis

For correlation analysis, the top 20 differential flavor compounds (VIP > 1) were selected. Spearman correlation coefficients were calculated to evaluate the associations between candidate genes, volatile flavors and fatty acids, with |R| > 0.7 and *p*-value < 0.05 considered indicative of a strong correlation.

### 2.6. Statistical Analysis

The organization and preliminary summarization of all experimental data were conducted with Excel 2021. Statistical analysis was then carried out in SPSS 27.0 (IBM Corp., Armonk, NY, USA). To evaluate intergroup differences between the H and L groups, the nonparametric Mann–Whitney U test was employed. A *p*-value < 0.05 was considered statistically significant, and a *p*-value < 0.01 was considered highly significant. Data from four biological replicates per group are presented as mean ± SD.

For the purposes of data visualization and statistical analysis, GraphPad Prism 9.3 and the Omicshare online platform (Version 2025, Guangzhou Genedenovo Technology Co., Ltd., Guangzhou, China) were applied [33]. Subsequent downstream analyses, such as heatmap analysis, principal component analysis (PCA), and orthogonal partial least squares-discriminant analysis (OPLS-DA), were also conducted through the tools available on the Omicshare platform.

## 3. Results

### 3.1. Fatty Acid Composition of Longissimus Dorsi Muscle

**Figure 1:**
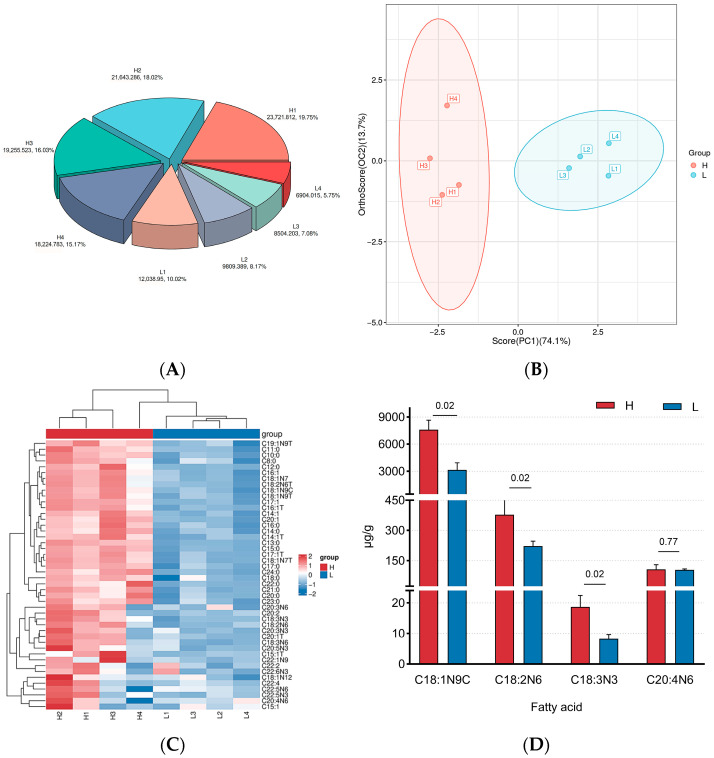
Composition and comparative analysis of fatty acids in DBS cattle with H and L groups fatty acid content. (**A**) The proportion of fatty acid content in the sample. Note: H represents the group with a high fatty acid content, and L represents the group with a low fatty acid content; (**B**) OPLS-DA score plotof fatty acid OPLS-DA; (**C**) Fatty acid composition correlation heatmap of samples. Note: horizontal represents sample name, vertical represents fatty acid information, group represents subgroup, red represents group H, and blue represents group L; (**D**) Analysis of the differences in key fatty acids affecting beef flavor. Note: horizontal represents the name of fatty acid, vertical represents the fatty acid content (μg/g).

Based on fatty acid content, the samples were divided into H and L groups (Figure 1A) (Supplementary Table S1). To evaluate the degree of variation between and within groups, PCA was performed on all samples. As shown in Figure 1B, the respective contributions of the first two principal components to the total variance were 75.5% and 13.2%. The results showed that a clear separation trend was observable between the two groups in the PCA score plot. Hierarchical cluster analysis confirmed that the 46 fatty acids formed two distinct clusters, which aligned with the experimental grouping (Figure 1C).

Fatty acids serve as crucial precursors for the formation of flavor compounds, and their content variations are key determinants of flavor development. Supplementary Table S1 shows that the fatty acid with the highest content in both groups was C18:1n9c, followed by C16:0 and C18:0. Differential analysis revealed that compared to the group L, the group H exhibited a significant increase (*p* < 0.05) in the content of most unsaturated fatty acids, with the exception of certain MUFAs such as C15:1, C18:1n12, and C20:1T, as well as PUFAs including C20:3n3, C20:3n6, C20:4n6, C22:1n9, C22:2, and C22:4. Notably, the concentrations of C18:1n9c, C18:2n6, and C18:3n3, which serve as key precursors of beef flavor, demonstrated significant differences between the H and L groups (*p* < 0.05). However, no significant difference was observed in C20:4n6 (*p* = 0.79) (Figure 1D).

### 3.2. Comparison of Volatile Flavor Compounds Among Breeds

#### 3.2.1. Types of Volatile Flavor Compounds and Overall Analysis for Sensory Flavor Characteristics

**Figure 2:**
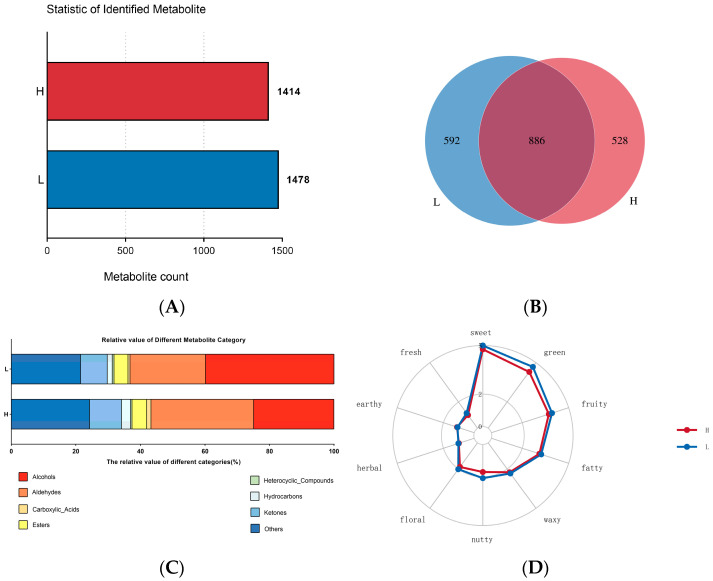
Analysis of VOCs and sensory flavor characteristics between H and L groups. (**A**) Differences in VOCs expression between the two groups; (**B**) Venn diagrams of the VOCs identified in the two groups; (**C**) Relative value of different VOCs; (**D**) Sensory flavor characterization with the designation in the outermost circle indicating the organoleptic flavor profile. The line indicates the detection frequency for the flavor substance (1–5, with a maximum of 5).

VOCs in the LD of DBS cattle were analyzed using comprehensive GC×GC-TOF MS. The results showed that 1414 and 1478 VOCs were identified in the H and L groups, respectively (Figure 2A) (Supplementary Table S2). Further comparative analysis revealed that 528 and 592 unique flavor compounds were exclusively detected in the H and L groups, respectively, while 886 compounds were shared between the two groups (Figure 2B) (Supplementary Table S3). These compounds were classified into eight categories: alcohols, aldehydes, acids, esters, heterocyclic compounds, hydrocarbons, ketones, and other flavor compounds (Figure 2C, Table 1). Notably, the relative contents of aldehydes and hydrocarbons were higher in the group H than in the L group, whereas the opposite trend was observed for alcohols, ketones, heterocyclic compounds, and other compounds (Figure 2C).

In addition, the sensory properties of VOCs in both groups were analyzed based on the Flavor DB database. The flavor sensory radar chart indicated a high similarity in flavor profiles between the two groups, with the overall predominant sensory trends being sweetness, green, fruity, and fatty notes (Figure 2D) (Supplementary Table S4). Among them, compounds exhibiting sweet characteristics were primarily alcohols, aldehydes, esters, and ketones, including 1-Heptanol, 2,4-Decadienal, (E,E)-, Ethyl Acetate, and 2,3-Butanedione. Compounds displaying green and fruity characteristics were mainly alcohols, aldehydes, and heterocyclic organic compounds, such as 1-Penten-3-ol, Hexanal, and Furan, 2-pentyl-. Meanwhile, compounds with fatty characteristics were predominantly aldehydes, including Hexanal, Heptanal, and Octanal.

#### 3.2.2. Analysis of Differential Volatile Flavor Compounds

**Figure 3:**
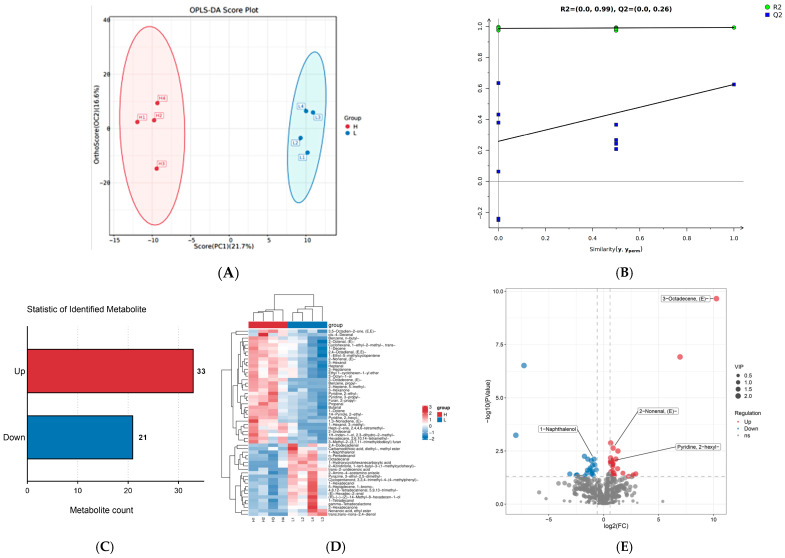

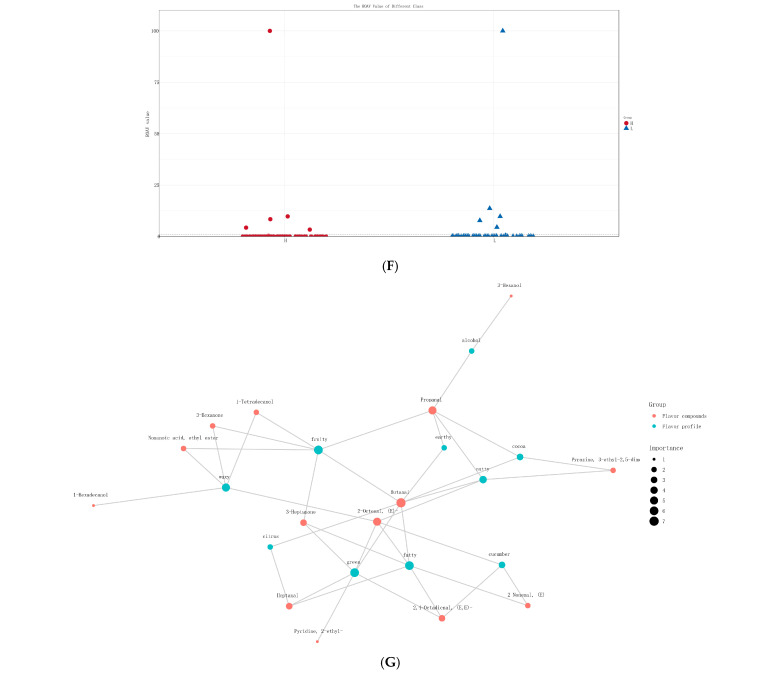
Identification of differences in VOCs between group H and group L. (**A**) OPLS-DA scores plot of two groups; (**B**) OPLS-DA model validation; (**C**) Statistical analysis of differential VOCs; (**D**) Overall cluster analysis conducted on differential volatile compounds. Note: The horizontal lines represent sample names, and the vertical lines represent VOCs information; (**E**) Volcanic diagrams of differential VOCs. Note: The abscissa is log_2_ (Fold Change) value, and the ordinate is −lg_10_ (*p*-value). Blue nodes represent down-regulated VOCs, red nodes represent up-regulated VOCs; (**F**) The Mahalanobis distance of each E-nose sensor: the farther from the red line (0, 0), the stronger the induction of this flavor; (**G**) Correlation network for the sensory flavor profile and flavor substances.

The OPLS-DA score plot (Figure 3A) of VOCs showed significant differences in the composition of volatile substances between groups H and L. A further permutation test showed that all blue Q^2^ points were lower than the original Q^2^ value of the model (Figure 3B), confirming the OPLS-DA model’s validity and the reliability. A total of 2006 flavor compounds registered with Chemical Abstracts Service (CAS, https://www.cas.org/cas-data/cas-registry, accessed on 16 March 2025) numbers were detected across both groups. Following filtration, 544 VOCs were retained. Based on the screening criteria of VIP value > 1 and *p*-value < 0.05, 54 differential flavor compounds were identified and screened out. Among these, 33 were up-regulated and 21 were down-regulated (Figure 3C).

Cluster heatmap and volcano plot analyses further illustrated the abundance patterns and statistical significance of these 54 differential compounds (Figure 3D,E). The assessment of each VOCs’ contribution to the overall flavor was performed using the ROAV. (E)-2-Nonenal was identified as the most impactful odorant in both groups (ROAV = 100), indicating its role as a key character impact compound (Figure 3F) (Supplementary Table S5). Subsequently, we constructed a correlation diagram of the interactions between VOCs and flavor (Figure 3G) to elucidate the relationship between the sensory flavor and flavor compounds in DBS cattle. The results indicated that their intense and distinctive flavors might arise from the relatively elevated levels of specific VOCs, such as Propanal, Butanal, (E)-2-Octenal, 3-Heptanone, and other compounds.

### 3.3. Gene Expression Profile

Transcriptome sequencing generated approximately 375 million raw reads (~53 Gb of data), with an average 46.8 million reads per sample. After filtering, an average of 97.34% (range: 97.03–97.70%) of the reads were retained as high-quality clean reads. Valid sequences aligned to the bovine reference genome ARS-UCD1.2, achieving an average alignment rate of 91.75% (Supplementary Table S6).

**Figure 4:**
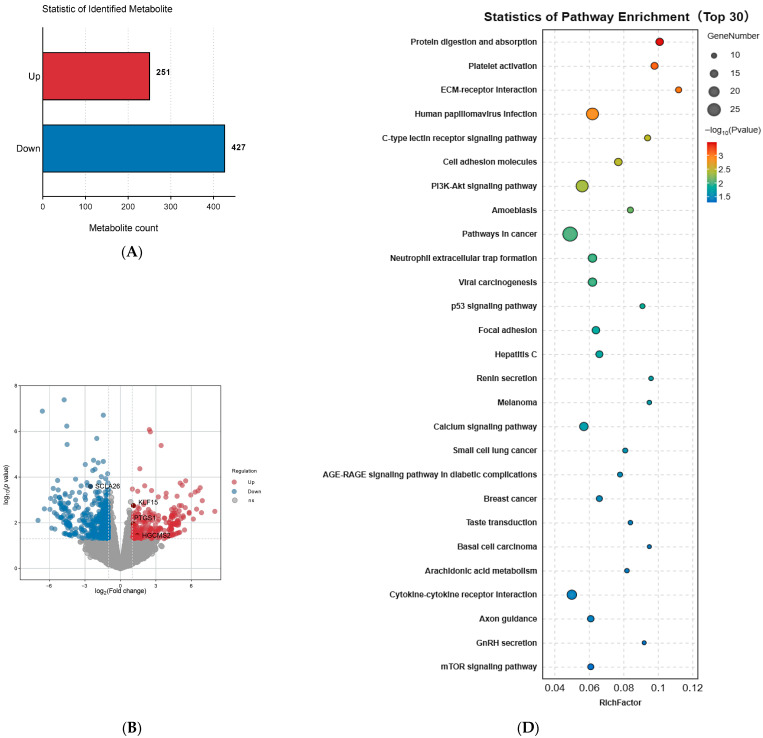

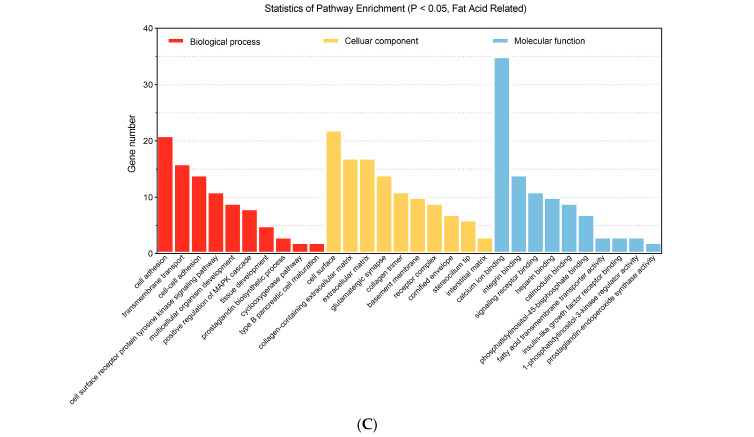
Transcriptome analysis between the two groups: (**A**) Statistical analysis of DEGs between the two groups; (**B**) Volcano maps of the DEGs between the two groups. Note: The abscissa is log2 (Fold Change) value, and the ordinate is −lg10 (*p*-value). Blue nodes represent down-regulated DEGs, red nodes represent up-regulated DEGs; (**C**) Fatty acid associated enriched GO terms; (**D**) The top 30 significant terms by KEGG enrichment analysis.

A total of 678 DEGs were identified through differential analysis, comprising 251 up-regulated genes and 427 down-regulated genes (Figure 4A). Volcano plot visualization revealed significant differences in gene expression patterns between the H and L groups (Figure 4B). Subsequently, GO and KEGG functional enrichment analyses were employed to determine the functions of DEGs in DBS cattle from the H and L groups. GO enrichment analysis identified 109 significantly enriched functional terms, primarily involved in fatty acid transmembrane transporter activity, the cyclooxygenase pathway, prostaglandin biosynthesis, and insulin-like growth factor receptor binding (Figure 4C) (Supplementary Table S7). Additionally, KEGG enrichment results demonstrated that these genes were significantly enriched in pathways such as arachidonic acid metabolism, the PI3K-Akt signaling pathway, the mTOR signaling pathway, and cytokine–cytokine receptor interactions (Figure 3D) (Supplementary Table S8). Based on the integrated functional annotation and pathway enrichment results, nine key candidate genes previously associated with fatty acid metabolism were selected. These genes are involved in distinct biological processes: fatty acid oxidation (*HMGCS2*, *ANGPTL8*), fatty acid transport (*SLC27A4*, *SLC27A6*), and lipogenesis and lipid metabolism (*KLF15*, *SGPL1*, *GPAM*, *ELOVL6*, *MOGAT3*).

### 3.4. Correlation Between Differentially Expressed Genes and Volatile Flavor Compounds

To investigate the genetic factors related to beef flavor, we assessed the correlations between fatty acid composition, gene expression, and key flavor compounds. Specifically, spearman correlation analysis was performed between the screened key DEGs and fatty acid content. Concurrently, the top 20 differential VOCs were identified based on VIP > 1 and subjected to Spearman correlation analysis with fatty acid content (Supplementary Table S9).

**Figure 5:**
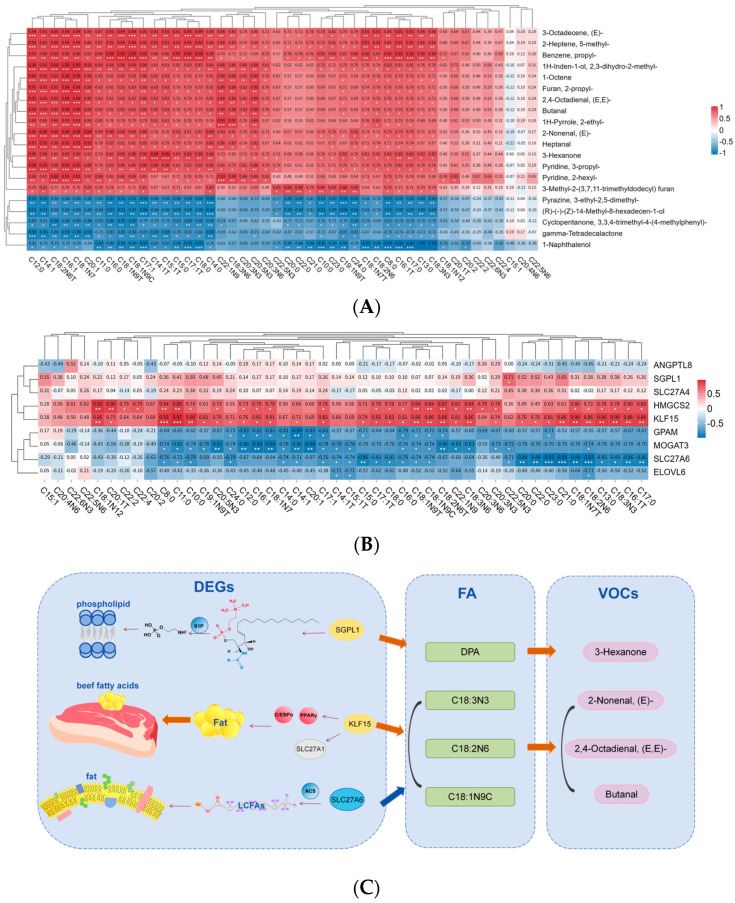
Correlation analysis between fatty acids and both the DEGs and the top 20 differential VOCs in the two groups: (**A**) Correlation analysis of fatty acids and VOCs (|R| ≥ 0.70, *p* < 0.05); Note: * indicates correlation coefficient |R| ≥ 0.70 and ≤0.79; ** indicates |R| ≥ 0.80 and ≤0.89; *** indicates |R| ≥ 0.90. (**B**) Correlation analysis of fatty acids and DEGs (|R| ≥ 0.70, *p* < 0.05); Note: * indicates correlation coefficient |R| ≥ 0.70 and ≤0.79; ** indicates |R| ≥ 0.80 and ≤0.89; *** indicates |R| ≥ 0.90. (**C**) Regulation mechanism diagram of DEGs and differential metabolites (fatty acid and VOCs). Note: Yellow ellipses denote up-regulated DEGs, while blue ellipses indicate down-regulated genes; gray ovals represent genes with insignificant differences; pink circles represent transcription factors, and blue circles denote enzymes. The green rectangle illustrates fatty acids, whereas the pink oval depicts volatile flavor substances.

The results indicated that the expression levels of *SGPL1*, *KLF15*, and *SLC27A6* genes were significantly correlated with the abundance of UFAs (C22:5n-3, C18:3n-3, C18:2n-6, C18:1n-9c), while these UFAs also exhibited significant associations with characteristic flavor compounds (3-Hexanone, 2-Nonenal, (E)-, 2,4-Octadienal, (E,E)-, and Butanal) (Figure 5A,B). This suggests that the accumulation of these fatty acids may be associated with upstream gene expression and further related to the formation of downstream flavor compounds (Figure 5C). Specifically, the expression of the *SGPL1* gene showed a significant positive correlation with C22:5n-3 (DPA) (*p* < 0.05), while DPA was significantly positively correlated with 3-Hexanone (*p* < 0.05). The expression of the *KLF15* gene exhibited significant positive correlations with three key precursor fatty acids of beef flavor, including C18:3n-3, C18:2n-6, and C18:1n-9c (*p* < 0.05). Moreover, these key fatty acids were significantly positively correlated with 2-Nonenal, (E)-, 2,4-Octadienal, (E,E)-, and Butanal (*p* < 0.05). The *SLC27A6* gene exhibited higher expression in the low-fatty acid group and showed a significant negative correlation with the aforementioned key precursor fatty acids (C18:3n-3, C18:2n-6, C18:1n-9c), presenting an opposite trend to *KLF15* (*p* < 0.05). However, we found that *SLC27A6* expression was also significantly positively correlated with the flavor compounds 2-Nonenal, (E)-, 2,4-Octadienal, (E,E)-, and Butanal (*p* < 0.05).

## 4. Discussion

Fatty acids are key factors that influence beef quality and nutritional composition. They not only determine the nutritional properties of beef but also significantly impact critical sensory attributes such as flavor, tenderness, and juiciness, as confirmed by numerous studies [34,35,36]. In this study, a total of 46 fatty acids were detected in LD samples obtained for high and low groups. To maximize detection efficiency with a limited sample size, an extreme phenotype sampling strategy was employed to divide the samples into H and L groups. Previous studies have demonstrated that extreme phenotype sampling can significantly improve statistical power and amplify intergroup differences [37]. Guey et al. conducted a simulation study and found that extreme sampling can double the estimated effect size and reduce the sample size required for replication studies by up to fourfold [38]. The analysis results revealed that the overall fatty acid content in group H was significantly higher than that in group L (*p* < 0.05), with C18:1n9c, C16:0, and C18:0 being the most prominent. This finding is consistent with previous studies on the fatty acid composition of other cattle breeds [39,40,41,42]. Studies have shown that oleic acid, linoleic acid, α-linolenic acid, and arachidonic acid can generate a series of aromatic compounds that contribute to the characteristic flavor of beef through reactions such as auto-oxidation or thermal oxidation [43]. To validate the rationality of the grouping, this study conducted differential analysis on these key flavor precursors. Notably, arachidonic acid levels did not differ significantly between the two groups. Arachidonic acid is a known precursor of flavor compounds such as hexanal [44], and its content has been reported to influence flavor in chicken meat [45]. The absence of a significant difference in arachidonic acid levels in DBS cattle may suggest a distinct regulatory mechanism maintaining its homeostasis, potentially contributing to the consistent flavor quality of this breed.

Fatty acids play a key role in the formation of aroma compounds, especially UFA, as core factors regulating meat flavor [46,47]. Oleic acid, the most abundant fatty acid in beef, has been shown to enhance flavor quality and overall palatability. Hoa et al. demonstrated by constructing in vitro meat-like model systems that C18:1n9c can significantly accelerate the Maillard reaction process, thereby promoting the formation of key flavor substances such as pyrazines (nutty aroma) and thiophenes (roasted aroma) [48]. Similarly, Navarro et al. showed that after adding oleic acid-rich macadamia nut oil to pig diet, the C18:1 content in the LD was significantly increased, and the caramel aroma and fat aroma intensity in sensory evaluation were significantly enhanced [49]. Linoleic acid and α-linolenic acid, recognized as essential fatty acids that the human body cannot synthesize, exhibited irreplaceable physiological functions [50]. Due to their high oxidative susceptibility, they undergo cleavage to produce volatile compounds such as Hexanal, 2,3-Octanedione and 1-Octen-3-ol, which directly contribute to characteristic meat flavors [51,52,53]. Furthermore, Chen et al. showed that regulating α-linolenic acid content in feed significantly increased the levels of aldehydes and alcohols in tilapia meat [54].

The composition characteristics of VOCs are significantly correlated with fatty acid profiles, while beef aroma characteristics ultimately depend on VOCs species, concentrations, odor thresholds and their synergistic or antagonistic effects [55,56]. The results showed that the relative contents of aldehydes, esters and hydrocarbons in group H were significantly higher than those in group L. This was consistent with the biochemical role of UFA as precursors for aldehyde synthesis [57]. Specifically, the unsaturated double bonds in UFA molecules are susceptible to oxidative cleavage, which produces aldehyde compounds that exhibit fatty and fruity flavor characteristics [58]. Aldehydes, mainly derived from lipid oxidation and Maillard reactions, are recognized as central contributors to beef fat aroma due to their low odor threshold and high aroma activity (fruit, fat, and nut aromas), and are essential for overall flavor formation [59]. It should be noted that the relative content of 2-Nonenal, (E)-and Octanal in group H was significantly higher than that in group L, and both of them were characteristic products of UFA thermal oxidation [60], so it was speculated that they might be the source of lipid aroma in group H. Esters typically impart floral aromas to food [61]. In this study, group H was enriched with Ethyl Acetate, Heptanoic acid, Ethyl Ester and Butanoic acid, which may originate from the esterification between alcohols and free fatty acids during lipid oxidation [62]. In contrast, group L exhibited higher relative contents of specific alcohols (1-Octen-3-ol, 1-Tetradecanol, and 1-Hexadecanol), carboxylic acids (Butanoic acid, 2-oxo- and Butanoic acid, 3-hydroxy-, ethyl ester), and heterocyclic compounds (Furan, 2-pentyl- and Pyrazine, ethyl-). Alcohols may be derived from UFA degradation or from aldehyde hydrogenation [63,64], while heterocyclic compounds such as furans are mainly formed by Maillard reactions during postmortem storage [65].

To identify the key flavor compounds, we performed a relative odor activity value (ROAV) analysis, where a ROAV of 100 represents the most significant contributor. The ROAV analysis identified (E)-2-Nonenal as the most significant contributor (ROAV = 100) in both sample sets, followed by 2,3-Butanedione, Heptanal, 2-Pentylfuran, and (E)-2-Octenal. Based on this, this study focused on the potential contribution of 2-nonenal, a ROAV peak substance, to the flavor of DBS cattle. It was found that (E)-2-Nonenal, as unsaturated aldehyde substance, could produce characteristic cucumber fragrance and fat fragrance through fatty acid peroxidation pathway [66,67], which has a core contribution to beef flavor [10,68]. It should be noted that Zhou et al. found that linoleic acid content was significantly positively correlated with (E)-2-Nonenal based on Huaxi cattle and Jia County red cattle, which was consistent with the Spearman correlation analysis results in this study [69].

Fatty acid composition is regulated by lipogenesis, a process under significant genetic control [70,71]. Three core genes positively correlated with fatty acids, namely *SGPL1*, *KLF15* and *SLC27A6*, were ultimately identified. *SGPL1* catalyzes the cleavage of sphingosine-1-phosphate to produce phosphoethanolamine, an important precursor for phospholipid synthesis [72]. Hydrolysis of phospholipids can release UFAs such as linoleic acid, arachidonic acid, and oleic acid, which are closely related to flavor formation [73]. Based on the above metabolic associations, we speculate that *SGPL1* may be involved in the accumulation of phospholipids through its association with the phospholipid synthesis pathway mediated by phosphoethanolamine. Thus, when hydrolysis occurs, in addition to releasing the known fatty acids mentioned above, it may also simultaneously release long-chain UFAs such as DPA. And these released fatty acids can further undergo oxidation, cracking and other reactions to generate volatile flavor substances. It is worth noting that DPA, as an intermediate in the metabolism of omega-3 fatty acids, can be converted into EPA (C20:5n-3)and DHA (C22:6n-3) through elongase action [74,75]. *KLF15*, a member of the *KLF* transcription factor family, plays a central regulatory role in lipogenesis [76]. Guo et al. confirmed that *KLF15* can enhance its transcriptional activity by directly binding to *KLF3* promoter, driving adipocyte differentiation and lipid deposition [77]. Zhao et al. further revealed that *KLF15* regulates fatty acid transport and muscle lipid metabolism by specifically binding to *SLC27A1* promoter [78]. In this study, *KLF15* exhibited high expression levels in group H, suggesting its potential involvement in fatty acid content in DBS cattle, possibly through effects on adipocyte differentiation, lipid deposition, or fatty acid transport processes. In contrast, *SLC27A6* gene (encoding a member of the fatty acid transporter family) showed differential expression patterns between H and L fatty acid groups, and its expression level was negatively correlated with *KLF15*. Given that the *SLC27* protein family is responsible for the transmembrane transport of long-chain fatty acids and the maintenance of lipid homeostasis [79], the low expression of this gene in the group H may indirectly affect the biosynthetic pathways of characteristic flavor compounds by altering specific fatty acids. These gene regulatory networks cooperate to shape the fatty acid profile and flavor quality of DBS cattle.

## 5. Conclusions

This study integrated fatty acid composition, VOCs, and transcriptomic data to systematically elucidate the genetic and metabolic basis of flavor formation in the LD of Dabieshan cattle. The results demonstrated significant enrichment of PUFAs such as C18:1n9c, C18:2n6 and C18:3n3 in the group H, with (E)-2-Nonenal as a key characteristic flavor compound among their oxidative derivatives. Transcriptomic analysis revealed that SGPL1 and KLF15 were positively correlated with these precursor fatty acids, whereas SLC27A6 showed a negative correlation. Further correlation analysis established significant associations between these fatty acids and characteristic flavor compounds (2-Nonenal, 3-Hexanone, (E,E)-2,4-Octadienal, and Butanal), suggesting a coordinated metabolic link among genes, fatty acids, and flavor. These findings provide critical molecular targets and a theoretical basis for genetic improvement of flavor traits in local yellow cattle breeds.

## Figures and Tables

**Table 1:** Classification of volatile flavor substances in the H and L groups of DBS cattle LD.

Group	Alcohols	Aldehydes	Carboxylic Acids	Esters	Heterocyclic Compounds	Hydrocarbons	Ketones	Others
H	118	69	35	77	232	195	111	577
L	136	68	35	77	235	179	130	616

## Data Availability

The datasets used and analyzed during the current study available from the corresponding author on academic request (L.X.). The data are not publicly available to preserve the privacy of the data.

## Supplementary Materials

The following supporting information can be downloaded at https://www.mdpi.com/article/10.3390/foods15081423/s1: Figure S1: Principal components analysis of gene expression; Table S1: Comparison of fatty acid composition (μg/g) between H and L groups of DBS cattle; Table S2: Total of 2006 VOCs were identified in the H and L groups of DBS cattle; Table S3: Unique differential VOCs identified in the H and L group of DBS cattle ; Table S4: Analysis of sensory flavor characteristics in H and L groups of DBS cattle ; Table S5: The relative odor activity (ROAV) VOCs identified in H and L groups; Table S6: Reads mapping summary of DBS cattle; Table S7: GO function annotation analysis of differentially expressed genes between H and L groups; Table S8: KEGG function enrichment analysis of differentially expressed genes between H and L groups; Table S9: Differential flavor compounds in Top 20 with VIP > 1.

## Abbreviations

DBS cattle: Dabieshan cattle; LD: longissimus dorsi; H: high groups; L: low groups; VOCs: volatile flavor compounds; SFAs: saturated fatty acids; MUFAs: monounsaturated fatty acids; ROAV: relative odor activity value; PUFAs: polyunsaturated fatty acids; GC×GC-TOF-MS: two-dimensional gas chromatography–time-of-flight mass spectrometry; OPLS-DA: orthogonal partial least squares-discriminant analysis; PCA: principal component analysis; DEGs: differentially expressed genes; GO: Gene Ontology; KEGG: Kyoto Encyclopedia of Genes and Genomes.
