# Supplementary material for: Integrative Analysis of Volatile Flavor Compounds and Transcriptome Reveals Underlying Mechanisms Linked to Fatty Acid Content in Dabieshan Cattle
Source: Foods. 2026 Apr 19;15(8):1423. doi: 10.3390/foods15081423 (PMC13114855; doi:10.3390/foods15081423)
Supplement: Supplementary file 1 [file foods-15-01423-s001.zip › Supplement Table S1.pdf]

**Supplementary Table S1 Comparison of fatty acid composition (μg/g) between H and L groups of DBS cattle**

| items | H | L | P-values |
|---|---|---|---|
| C8:0 | 1.71±0.27 | 0.89±0.31 | 0.02 |
| C10:0 | 12.39±1.19 | 5.32±1.2 | 0.02 |
| C11:0 | 1.44±0.06 | 1.19±0.04 | 0.02 |
| C12:0 | 28±5.58 | 11.41±0.75 | 0.02 |
| C13:0 | 3.46±0.13 | 2.29±0.07 | 0.02 |
| C14:0 | 714.16±98.58 | 322.69±76.67 | 0.02 |
| C14:1 | 187.57±26.35 | 60.28±19.39 | 0.02 |
| C14:1T | 8.67±1.21 | 3.48±0.72 | 0.02 |
| C15:0 | 77.43±5.17 | 27.03±4.68 | 0.02 |
| C15:1 | 6.55±0.86 | 6.21±0.36 | 0.77 |
| C15:1T | 2.14±0.54 | 1.41±0.07 | 0.02 |
| C16:0 | 6761.61±988.72 | 3058.81±726.68 | 0.02 |
| C16:1 | 802.65±129.52 | 272.47±94.62 | 0.02 |
| C16:1T | 48.18±7.65 | 17.97±2.93 | 0.02 |
| C17:0 | 151.48±9.4 | 61.58±20.34 | 0.02 |
| C17:1 | 97.1±12.8 | 33.87±7.03 | 0.02 |
| C17:1T | 22.47±2.21 | 9.26±2.07 | 0.02 |
| C18:0 | 2874.59±204.34 | 1547.2±660.38 | 0.02 |

| | | | |
|---|---|---|---|
| C18:1N12 | 161.29±51.41 | 112.28±33.97 | 0.15 |
| C18:1N7 | 251.36±52.83 | 99.5±27.22 | 0.02 |
| C18:1N7T | 201.28±23.45 | 65.84±21.42 | 0.02 |
| C18:1N9C | 7588.93±1063.84 | 3138.06±800.77 | 0.02 |
| C18:1N9T | 44.23±6.32 | 21.01±4.19 | 0.02 |
| C18:2N6 | 378.43±72.26 | 222.12±24.74 | 0.02 |
| C18:2N6T | 14.88±2.7 | 6.85±1.13 | 0.02 |
| C18:3N3 | 18.69±3.73 | 8.28±1.4 | 0.02 |
| C18:3N6 | 5.87±0.75 | 4.54±0.34 | 0.04 |
| C19:1N9T | 5.14±0.33 | 3.56±0.42 | 0.02 |
| C20:0 | 19.84±3.24 | 8.73±2.53 | 0.02 |
| C20:1 | 22.55±3.18 | 9.77±1.5 | 0.02 |
| C20:1T | 1.01±0.6 | 0.24±0.14 | 0.19 |
| C20:2 | 7.01±1.56 | 4.97±0.29 | 0.15 |
| C20:3N3 | 2.78±0.44 | 2.26±0.18 | 0.08 |
| C20:3N6 | 26.22±4.13 | 22.67±2.64 | 0.15 |
| C20:4N6 | 107.56±22.43 | 104.35±5.2 | 0.77 |
| C20:5N3 | 5.28±0.64 | 4.39±0.24 | 0.04 |
| C21:0 | 3.79±0.24 | 2.74±0.2 | 0.02 |
| C22:0 | 3.28±0.74 | 1.2±0.2 | 0.02 |

| | | | |
|---|---|---|---|
| C22:1N9 | 1.46±1.04 | 0.32±0.23 | 0.04 |
| C22:2 | 4.27±0.53 | 3.98±0.36 | 0.39 |
| C22:4 | 7.64±5.4 | 3.6±1.19 | 0.39 |
| C22:5N3 | 13.8±3.06 | 10.23±0.78 | 0.04 |
| C22:5N6 | 5.2±1.97 | 4.67±0.42 | 0.77 |
| C22:6N3 | 2.55±0.79 | 1.95±0.75 | 0.39 |
| C23:0 | 2.6±0.28 | 1.36±0.33 | 0.02 |
| C24:0 | 2.81±0.31 | 1.3±0.16 | 0.02 |
| ΣSFA | 10658.6±1245.21 | 5053.73±1373.66 | 0.02 |
| ΣUFA | 10052.76±1444.36 | 4260.41±931.09 | 0.02 |
| ΣMUFA | 9452.58±1345.29 | 3855.54±948.46 | 0.02 |
| ΣPUFA | 600.18±113.25 | 404.87±26.03 | 0.02 |

[a]Note: *P*-values from two-tailed Mann-Whitney U test (H vs. L). $p < 0.05$: statistically significant; $p \geq 0.05$: not significant. Data: mean ± SD (n = 4 per group). Abbreviations: T, trans isomer; ΣUFA, ΣMUFA, ΣPUFA as defined.
