# Supplementary material for: Integrative Analysis of Volatile Flavor Compounds and Transcriptome Reveals Underlying Mechanisms Linked to Fatty Acid Content in Dabieshan Cattle
Source: Foods. 2026 Apr 19;15(8):1423. doi: 10.3390/foods15081423 (PMC13114855; doi:10.3390/foods15081423)
Supplement: Supplementary file 1 [file foods-15-01423-s001.zip › Supplement Table S7-8.pdf]

Supplement Table S7. GO function annotation analysis of differentially expressed genes between H and L groups.

Group	Category	GO ID	Description	P value
H vs L	BP	GO:0007155	cell adhesion	2.66066E-05
	BP	GO:0055085	transmembrane transport	0.025895102
	BP	GO:0007169	cell surface receptor protein tyrosine kinase signaling pathway	6.37425E-05
	BP	GO:0098609	cell-cell adhesion	7.4839E-05
	BP	GO:0009888	tissue development	0.004417865
	BP	GO:0043410	positive regulation of MAPK cascade	0.021017676
	BP	GO:0007275	multicellular organism development	0.02250259
	BP	GO:0001516	prostaglandin biosynthetic process	0.041268222
	BP	GO:0019371	cyclooxygenase pathway	0.046238081
	BP	GO:0072560	type B pancreatic cell maturation	0.046238081
	CC	GO:0062023	collagen-containing extracellular matrix	7.0391E-08
	CC	GO:0031012	extracellular matrix	7.14808E-07
	CC	GO:0005581	collagen trimer	3.24264E-06
	CC	GO:0001533	cornified envelope	1.44923E-05
	CC	GO:0032426	stereocilium tip	1.89913E-05
	CC	GO:0005604	basement membrane	2.41237E-05
	CC	GO:0009986	cell surface	6.37522E-05
	CC	GO:0098978	glutamatergic synapse	0.000860273
	CC	GO:0005614	interstitial matrix	0.019723781
	CC	GO:0043235	receptor complex	0.022079067
	MF	GO:0005178	integrin binding	1.74322E-06
	MF	GO:0005509	calcium ion binding	1.80222E-05
	MF	GO:0008201	heparin binding	0.001751031
	MF	GO:0005546	phosphatidylinositol-4,5-bisphosphate binding	0.001963925
	MF	GO:0015245	fatty acid transmembrane transporter activity	0.009652608
	MF	GO:0005516	calmodulin binding	0.014926041
	MF	GO:0005102	signaling receptor binding	0.015435068
	MF	GO:0005159	insulin-like growth factor receptor binding	0.019790037
	MF	GO:0046935	1-phosphatidylinositol-3-kinase regulator activity	0.042922356
	MF	GO:0004666	prostaglandin-endoperoxide synthase activity	0.044051356

[a]Note:*P*-values were calculated using the modified Fisher's exact test (EASE score) in DAVID.
A p-value < 0.05 indicates statistically significant enrichment.

Supplement Table S8. KEGG function enrichment analysis of differentially expressed genes between H and L groups.

Group	KEGG ID	Description	P value
H vs L	bta04974	Protein digestion and absorption	0.000213565
	bta04611	Platelet activation	0.000546877
	bta04512	ECM-receptor interaction	0.000716844
	bta05165	Human papillomavirus infection	0.001756504
	bta04514	Cell adhesion molecules	0.002357697
	bta04625	C-type lectin receptor signaling pathway	0.002474335
	bta04151	PI3K-Akt signaling pathway	0.002579928
	bta05160	Hepatitis C	0.004319323
	bta05146	Amoebiasis	0.005360826
	bta05200	Pathways in cancer	0.005363496
	bta04933	AGE-RAGE signaling pathway in diabetic complications	0.006843564
	bta05164	Influenza A	0.007965752
	bta04115	p53 signaling pathway	0.00975075
	bta04510	Focal adhesion	0.01033906
	bta04020	Calcium signaling pathway	0.013846294
	bta04924	Renin secretion	0.01476772
	bta05218	Melanoma	0.015713283
	bta05222	Small cell lung cancer	0.018872964
	bta05224	Breast cancer	0.023079339
	bta04060	Cytokine-cytokine receptor interaction	0.024611391
	bta04742	Taste transduction	0.026164393
	bta04360	Axon guidance	0.027302257
	bta05217	Basal cell carcinoma	0.028866006
	bta00590	Arachidonic acid metabolism	0.028991899
	bta05143	African trypanosomiasis	0.0295428
	bta04929	GnRH secretion	0.032474853
	bta04064	NF-kappa B signaling pathway	0.037085962

[a]Note:*P*-values were calculated using the modified Fisher's exact test (EASE score) in DAVID. A p-value < 0.05 indicates statistically significant enrichment.
