# Supplementary figures and images for: Integrative Analysis of Volatile Flavor Compounds and Transcriptome Reveals Underlying Mechanisms Linked to Fatty Acid Content in Dabieshan Cattle
Source: Foods. 2026 Apr 19;15(8):1423. doi: 10.3390/foods15081423 (PMC13114855; doi:10.3390/foods15081423)

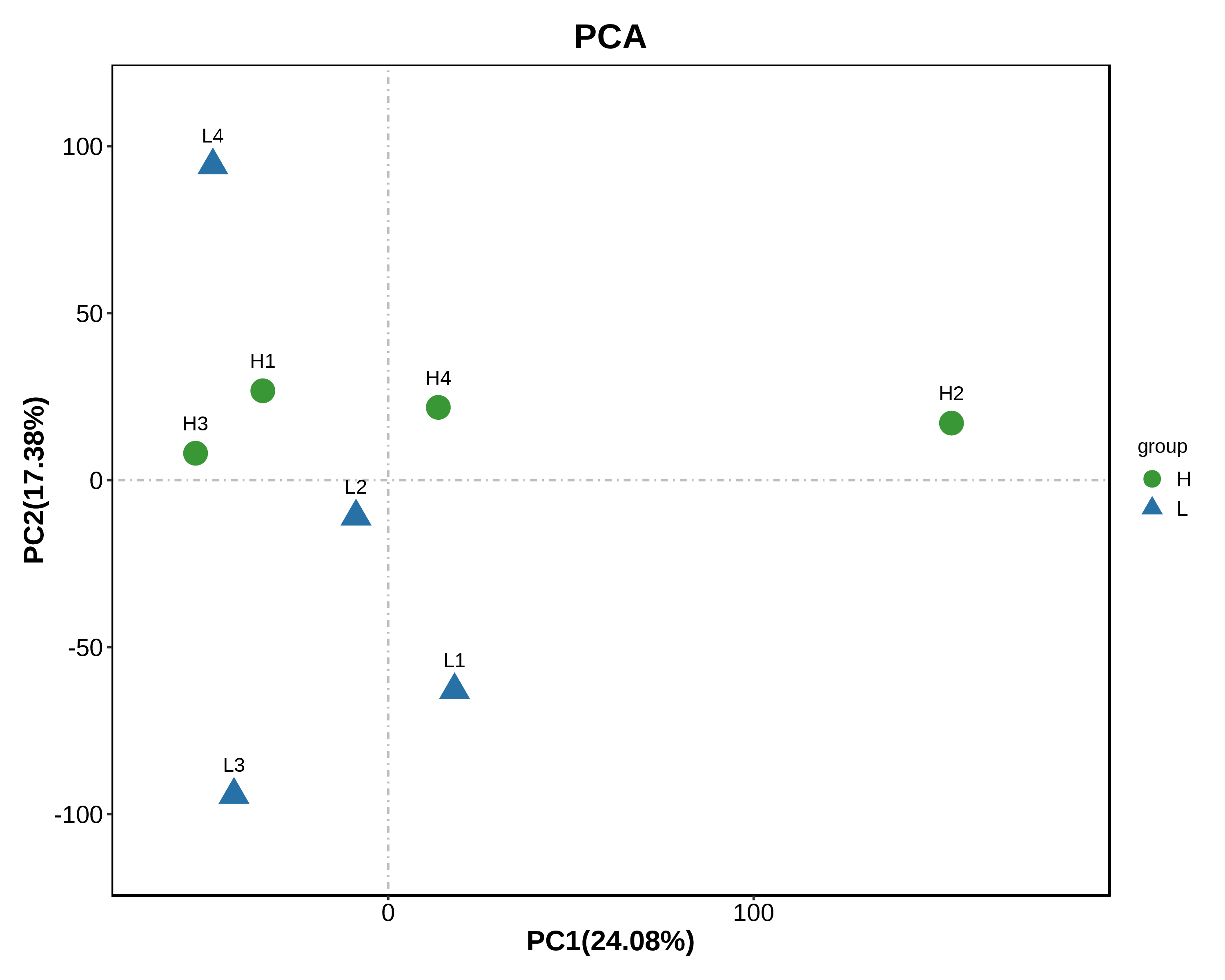

Supplement: Supplementary file 1 [file foods-15-01423-s001.zip › Supplementary Figure S1.png]
